# Long non‐coding RNA screening and identification of potential biomarkers for type 2 diabetes

**DOI:** 10.1002/jcla.24280

**Published:** 2022-03-07

**Authors:** Qi Ma, Li Wang, Zhiqiang Wang, Yinxia Su, Qinqin Hou, Qiushuang Xu, Ren Cai, Tingting Wang, Xueli Gong, Qizhong Yi

**Affiliations:** ^1^ Xinjiang Key Laboratory of Metabolic Disease Clinical Medical Research Institute The First Affiliated Hospital of Xinjiang Medical University Urumqi China; ^2^ Kuntuo Medical Research and Development Company Shanghai China; ^3^ 74790 Hospital of Public Health Xinjiang Medical University Urumqi China; ^4^ Department of pathology Fudan university Shanghai cancer center Shanghai China; ^5^ Psychological Medicine Center The First Affiliated Hospital of Xinjiang Medical University Urumqi China; ^6^ Specimen Bank of Xinjiang Key Diseases Clinical Medical Research Institute The First Affiliated Hospital of Xinjiang Medical University Urumqi China; ^7^ School of Nursing & Health Management Shanghai University of Medicine & Health Sciences Shanghai China; ^8^ 74790 Department of Pathophysiology School of Basic Medical Science Xinjiang Medical University Urumqi China

**Keywords:** biological markers, expression regulation, lncRNA, microarray analysis, type 2 diabetes

## Abstract

**Background:**

To investigate new lncRNAs as molecular markers of T2D.

**Methods:**

We used microarrays to identify differentially expressed lncRNAs and mRNAs from five patients with T2D and paired controls. Through bioinformatics analysis, qRT‐PCR validation, ELISA, and receiver operating characteristic (ROC) curve analysis of 100 patients with T2D and 100 controls to evaluate the correlation between lncRNAs and T2D, and whether lncRNAs could be used in the diagnosis of T2D patients.

**Results:**

We identified 68 and 74 differentially expressed lncRNAs and mRNAs, respectively. The top five upregulated lncRNAs are ENST00000381108.3, ENST00000515544.1, ENST00000539543.1, ENST00000508174.1, and ENST00000564527.1, and the top five downregulated lncRNAs are TCONS_00017539, ENST00000430816.1, ENST00000533203.1, ENST00000609522.1, and ENST00000417079.1. The top five upregulated mRNAs are Q59H50, CYP27A1, DNASE1L3, GRIP2, and lnc‐TMEM18‐12, and the top five downregulated mRNAs are GSTM4, PODN, GLYATL2, ZNF772, and CLTC. Examination of lncRNA‐mRNA interaction pairs indicated that the target gene of lncRNA XR_108954.2 is *E2F2*. Multiple linear regression analysis showed that XR_108954.2 (*r* = 0.387, *p* < 0.01) and *E2F2* (*r* = 0.368, *p* < 0.01) expression levels were positively correlated with glucose metabolism indicators. Moreover, *E2F2* was positively correlated with lipid metabolism indicators (*r* = 0.333, *p* < 0.05). The area under the ROC curve was 0.704 (95% CI: 0.578–0.830, *p* = 0.05) for lncRNA XR_108954.2 and 0.653 (95% CI: 0.516–0.790, *p* = 0.035) for *E2F2*.

**Conclusions:**

This transcriptome analysis explored the aberrantly expressed lncRNAs and identified *E2F2* and lncRNA XR_108954.2 as potential biomarkers for patients with T2D.

## Graphical abstract

Scatter plot analysis of lncRNAs (A) and mRNAs (B) expression. We identified differentially expressed 68 lncRNAs from the peripheral blood cells (44 up‐regulated and 24 down‐regulated) and 74 differentially expressed mRNAs (56 up‐regulated and 18 down‐regulated).

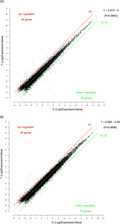

## 1 INTRODUCTION

Patients with type 2 diabetes (T2D) have continuously elevated circulating glucose levels, which is the pathological basis of various diseases.^1^, ^2^ Patients with T2D are at a higher risk of heart disease and cerebrovascular disease, accompanied by a higher risk of low‐position amputation than healthy individuals.^3^, ^4^ A large proportion of public medical resources, greater than that needed for patients with hypertension, stroke, and coronary artery disease combined, is required to care for patients with T2D.^5^, ^6^ T2D is an insidious disease, and delayed diagnosis and treatment lead to a failure in controlling the blood glucose levels. Therefore, new biomarkers and diagnostic approaches are urgently required for clinical therapy.

In recent years, intensive studies pertaining to long non‐coding RNAs (lncRNAs) have shown that they are widely involved in biological processes.^7^ lncRNAs participate in the regulation of gene expression by binding to homologous DNA, RNA, and a variety of proteins.^8^ lncRNAs have also been associated with many human diseases, including cancer,^9^ cardiovascular disease,^10^ diabetes,^11^ and mental disorders.^12^ Gao et al. found that compared with the control groups, the expression of lncRNA H19 was significantly reduced in patients with T2D as well as in insulin‐resistant mice.^13^ Our previous research has shown that lncRNA MEG3 is significantly downregulated in endothelial cells cultured in high glucose concentrations. Additionally, MEG3 knockdown promotes endothelial cell proliferation and reduces apoptosis at high glucose concentrations.^14^ Moreover, there is increasing evidence suggesting that lncRNAs may function as novel diagnostic and therapeutic targets for many diseases.^15^, ^16^ Therefore, systematic identification of differentially expressed lncRNAs in T2D, elucidation of the underlying mechanism, and evaluation of their clinical significance are necessary in patients with T2D.

In the present study, we analyzed aberrantly expressed lncRNAs in patients with T2D and performed functional enrichment and metabolic pathway analysis to explore their pathogenesis. One of the lncRNA‐mRNA pairs was chosen to validate the observed expression patterns, and the ROC curve was used to provide references for the diagnosis and treatment of T2D.

## 2 MATERIALS AND METHODS

### 2.1 Participants

In the screening stage, five patients with T2D and five healthy controls were recruited for the analysis of differentially expressed lncRNA/mRNA using microarray assay. Then, 100 patients and paired controls were assessed for validation by qRT‐PCR. T2D was evaluated according to the World Health Organization definition: fasting plasma glucose ≥7.0 mmol/l; 2‐h post‐load venous plasma glucose ≥11.1 mmol/l. The exclusion criteria were as follows: type 1 diabetes mellitus (T1DM) with chronic diseases, family history of T2D, and other types of chronic diseases. All subjects were enrolled at the First Clinical Affiliated Hospital of Xinjiang Medical University (Urumqi, China) from October 2016 to February 2017.

### 2.2 Biochemical indicator detection

Fasting plasma glucose (FPG), total cholesterol (TC), high‐density lipoprotein (HDL), low‐density lipoprotein (LDL), glycated serum protein (GSP), and triglyceride (TG) levels were measured in the Laboratory Medicine of the First Affiliated Hospital of Xinjiang Medical University.

### 2.3 Sample preparation and RNA purification

Density gradient centrifugation was used to purify peripheral blood mononuclear cells (PBMCs) from the blood obtained from the patients. TRIzol (Invitrogen, Carlsbad, CA, USA) was used to extract total RNA according to the manufacturer's instructions and quantified using a NanoDrop spectrophotometer (ND‐2000, NanoDrop Products, Wilmington, DE, USA).

### 2.4 Microarray assay

An Agilent Microarray (V4.0, CapitalBio; Beijing, China) was used to analyze the samples from the screening stage. A total of 41,000 lncRNAs and 34,000 mRNAs were evaluated by each slide (4 × 180 K format). Following the manufacturer's standard protocols, the samples were tagged, hybridized, and eluted. This process included reverse transcription of total RNA into double‐stranded cDNA, synthesis of cRNA, synthesis of cDNA by cRNA reverse transcription and fragmentation, hybridization, and cleaning with the chip after fluorescent labeling.^10^ The Agilent chip scanner (G2565CA) was used to obtain hybrid pictures.

Data were normalized and analyzed using GeneSpring GX software (Agilent Technologies, USA) and visualized using Agilent software (Feature Extraction, version 11.0.1.1). Differentially expressed genes were identified as those with fold change (FC) > 2 and *p*‐value <0.05.

### 2.5 Bioinformatics analysis

Gene ontology (GO) analysis was used to analyze gene function. Kyoto Encyclopedia of Genes and Genomes (KEGG) was used to identify biological pathways. The lncRNA‐mRNA co‐expression network was constructed using Cytoscape (v3.1.1, National Institute of General Medical Sciences, Washington, DC, USA). lncRNA‐mRNA co‐expression prediction analysis (correlation >0.99 or correlation <−0.99, *p* < 0.05) was used to screen the lncRNA‐mRNA pairs within a 10‐kb genomic location and similar sequences in the 3'‐UTR.

### 2.6 qRT‐PCR validation

Differentially expressed lncRNAs and mRNAs were determined using qRT‐PCR. Briefly, total RNA (1 µg) was extracted following the manufacturer's instructions. PCR was performed on an ABI 7500 System (Applied Biosystems, Carlsbad, CA, USA) using SYBR (TaKaRa Bio, Dalian, China). The 2^−△△CT^ method was used to calculate the fold change, and β‐actin was used for normalization. All experiments were repeated thrice.

### 2.7 Detection of plasma E2F2 protein

Peripheral blood (2 ml) was collected in EDTA tubes from each participant, and plasma was separated by centrifugation. Plasma concentrations of E2F2 were measured using an ELISA kit (Shanghai Hengyuan Biological Technology Co., Ltd., China) following the manufacturer's protocol.

### 2.8 Statistical analysis

All statistical analyses were performed using SPSS (v22.0, Chicago, IL, USA) and GraphPad Prism (v5.0, GraphPad Software Inc., San Diego, CA, USA). *χ* ^2^ and independent *t* tests were used to determine the differences between the population characteristics of patients with T2D and controls. The Mann–Whitney U test was used for abnormally distributed data. Significant GO terms and KEGG pathways were screened using Fisher's exact test. The lncRNA‐mRNA co‐expression network and clinical significance were constructed using multiple linear regression. Statistical significance was set at *p* ≤ 0.05. The specificity and sensitivity of lncRNAs were determined using receiver operating characteristic (ROC) curves.

## 3 RESULTS

### 3.1 Participant demographics and clinical characteristics

A total of 105 patients with T2D and paired controls were enrolled in our study with two stages. The χ^2^‐test and t test results revealed no significant differences between the T2D and control groups in terms of age or sex distribution, except for FPG. In stage one, FPG was an average of 10.85 mmol/l in patients with T2D and 4.87 mmol/l in the control group. In stage two, FPG was an average of 9.49 mmol/l in patients with T2D and 4.96 mmol/l in the control subjects (Table 1).

**TABLE 1:** Clinical characteristics of patients with T2D and controls

	T2D	Controls	Comparison	
			Statistics	*p*‐value
Stage One	*n* = 5	*n* = 5		
Age (y)	53.00±4.06	57.00±5.74	*t* = −1.271	0.239
Sex (Female/Male)	2/3	2/3	*χ* ^2^ = 0.000	1.000
FPG (mmol/l)	10.85±2.52	4.87±0.63	*t* = 6.514	<0.0001
Ethnicity	Han	Han		
Stage Two	*n* = 100	*n* = 100		
Age (y)	57.58±8.64	55.11±6.57	*t* = −1.489	0.140
Sex (Female/Male)	43/57	39/61	*χ^2^* = 0.331	0.565
FPG (mmol/l)	9.49±2.53	4.96±0.40	*t* = −11.863	<0.0001
Ethnicity	Han	Han		

### 3.2 lncRNA and mRNA microarray expression profiling

In stage one, ten blood samples were used for microarray profiling. Screening data can be obtained from the Gene Expression Omnibus (GEO) database (https://www.ncbi.nlm.nih.gov/geo/query/acc.cgi?acc=GSE163980).

**FIGURE 1:**
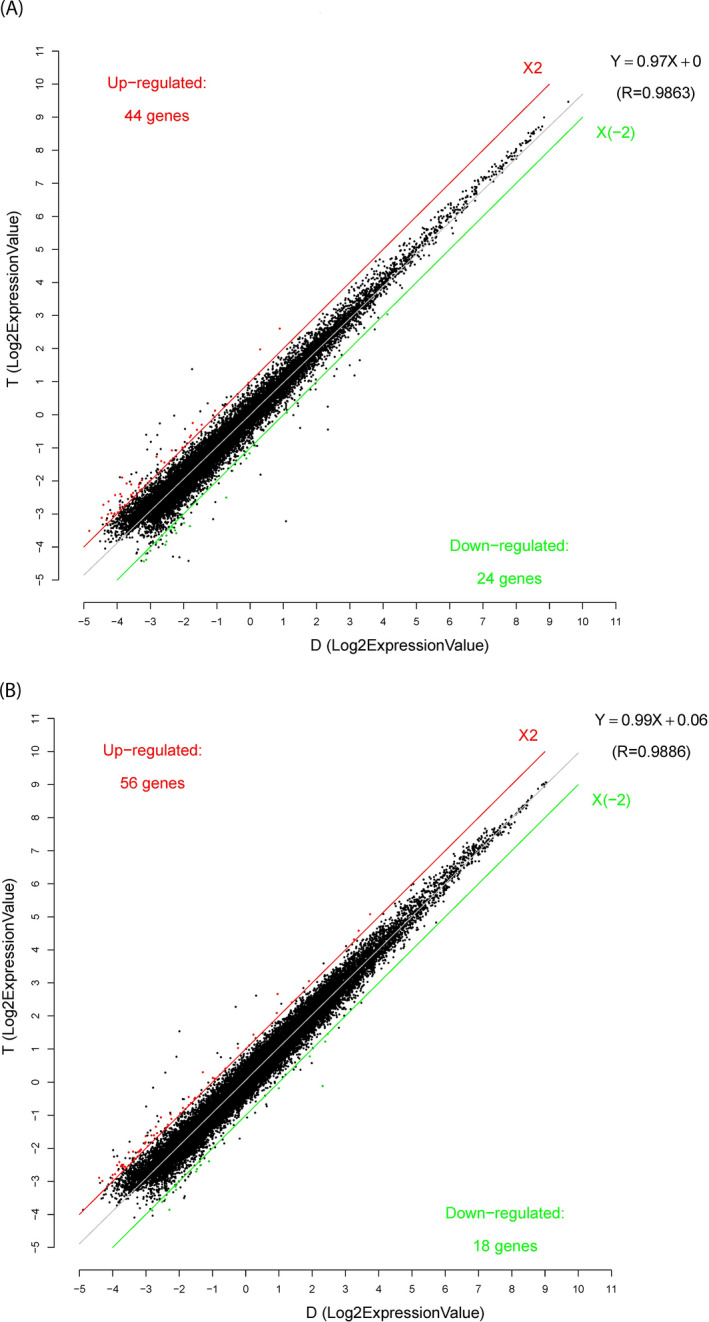
Scatter plot analysis of lncRNAs (A) and mRNAs (B) expression

We identified 68 differentially expressed lncRNAs (44 upregulated and 24 downregulated lncRNAs; FC > 2.0, *p* < 0.05) and 74 differentially expressed mRNAs (56 upregulated and 18 downregulated mRNAs; FC > 2.0, *p* < 0.05) from the peripheral blood cells (Figure 1). The top five differentially expressed lncRNAs and mRNAs are listed in Table 2.

**TABLE 2:** LncRNAs and mRNAs differentially expressed in T2D and control groups

Seqname	FC	*P*	Regulation	Type	chr
ENST00000381108.3	3.855	0.036	Up	lncRNA	chrX: 3771050‐3781615
ENST00000515544.1	3.269	0.010	Up	lncRNA	chr4: 80413746‐80497612
ENST00000539543.1	3.216	0.001	Up	lncRNA	chr1: 148928344‐148951595
ENST00000508174.1	3.179	0.017	Up	lncRNA	chr4: 80413569‐80497614
ENST00000564527.1	3.105	0.002	Up	lncRNA	chr15: 99679521‐99685575
TCONS_00017539	2.952	0.032	Down	lncRNA	chrX: 119264663‐119269975
ENST00000430816.1	2.618	0.035	Down	lncRNA	chr9: 138506141‐138507354
ENST00000533203.1	2.578	0.005	Down	lncRNA	chr11: 36408070‐36409800
ENST00000609522.1	2.498	0.007	Down	lncRNA	chr7: 38365670‐38369244
ENST00000417079.1	2.402	0.002	Down	lncRNA	chr13: 30916596‐30939898
Q59H50	3.253	0.042	Up	mRNA	chr21: 046327961‐046327902
CYP27A1	2.995	0.019	Up	mRNA	chr2: 219679951‐219680010
DNASE1L3	2.856	0.017	Up	mRNA	chr3: 58178414‐58178355
GRIP2	2.797	0.012	Up	mRNA	chr3: 14530795–14530736
lnc‐TMEM18‐12	2.631	0.017	Up	mRNA	chr2: 945382‐945323
GSTM4	5.397	0.027	Down	mRNA	chr1: 110201628‐110201686
PODN	2.963	0.008	Down	mRNA	chr1: 53547711‐53547770
GLYATL2	2.449	0.041	Down	mRNA	chr11: 58602251‐58602192
ZNF772	2.324	0.017	Down	mRNA	chr19: 57984876‐57984817
CLTC	2.268	0.001	Down	mRNA	chr17: 57774258‐57774317

Abbreviation: FC: fold changes; up: upregulation; down: downregulation.

### 3.3 Bioinformatics analysis

**FIGURE 2:**
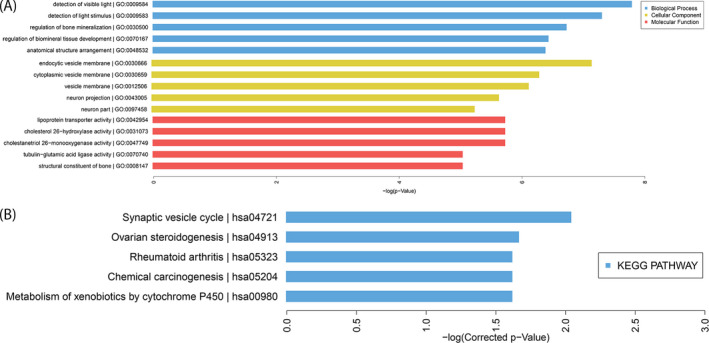
Gene ontology analysis (A) and Kyoto Encyclopedia of Genes and Genomes pathway analysis (B) of differentially expressed mRNAs

Genes perform their biological functions through coordination. This is especially true for complex diseases, such as T2D, which may be the result of a phenotypic difference caused by mutations in multiple genes.^17^ To investigate enriched genes in biological processes (BP), cellular components (CC), and molecular functions (MF), GO analysis was performed with the differentially expressed mRNAs. The top five most significant GO terms for each module based on the FDR ≤ 0.05 (Bonferroni correction) and *P*‐value are listed in Figure 2A. The most enriched BP term was detection of visible light. The most enriched CC term was endocytic vesicle membrane, while the most enriched MF term was lipoprotein transporter activity.

Additionally, we analyzed the significant pathways associated with consensus mutations in T2D patients using KEGG. The top five pathways are represented in a histogram image in Figure 2B; the most highly enriched pathway was observed to be the synaptic vesicle cycle.

### 3.4 Co‑expression analysis and target prediction

**FIGURE 3:**
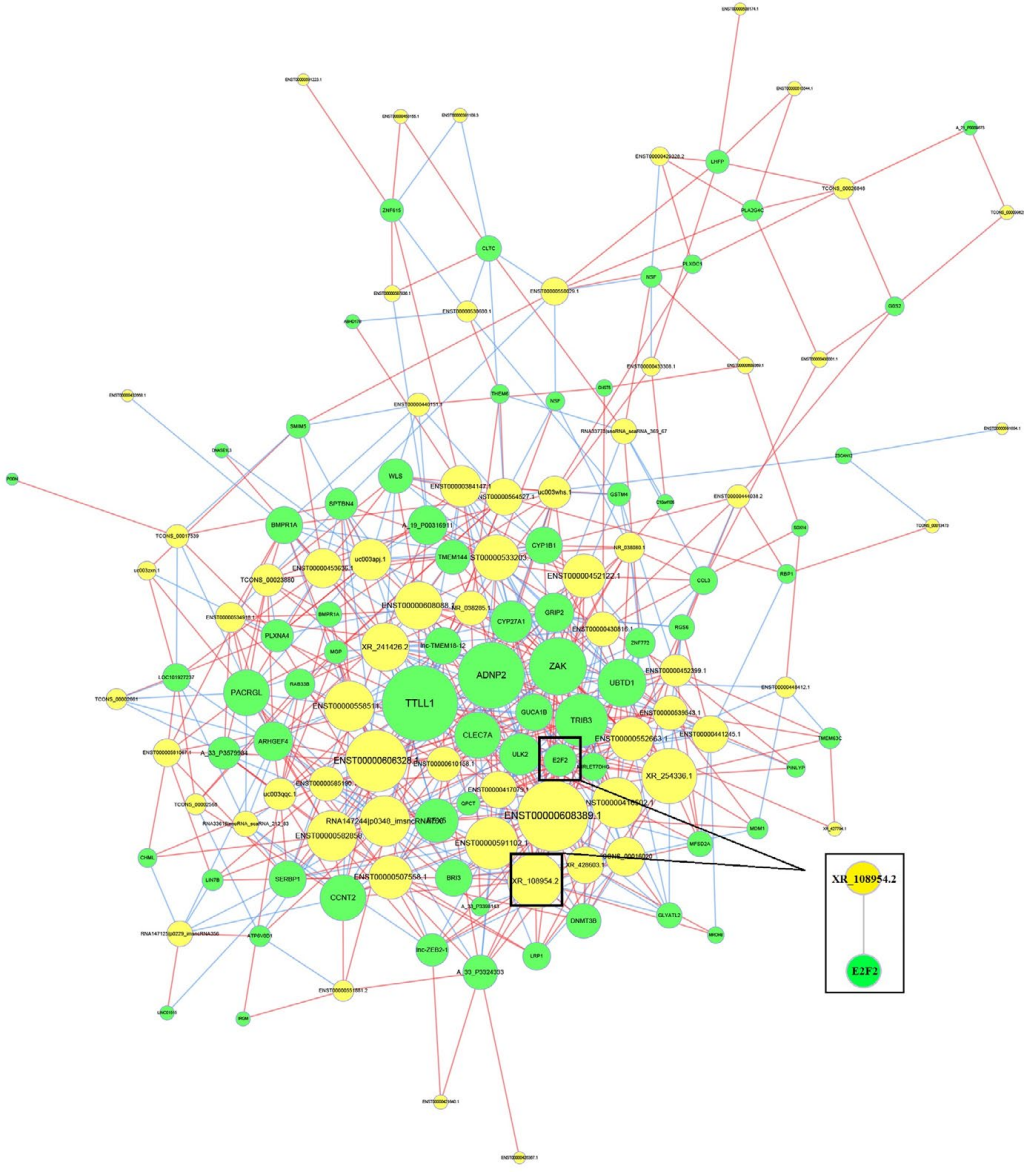
Co‐expression analysis of lncRNA‐mRNA and lncRNA target gene prediction. Yellow nodes: lncRNAs; green nodes: mRNAs. Red lines: positive correlation; blue lines: negative correlation

Next, we constructed lncRNA‐mRNA co‐expression network to investigate the correlation between differentially expressed lncRNAs and targeted mRNAs. Co‐expression analysis, based on mathematical correlation, was used to identify lncRNA‐mRNA pairs with similar expression profiles. The top 1000 pairs with the highest levels of correlation were selected, and Cytoscape software was used to draw the network diagram (Figure 3). Based on lncRNA and mRNA co‐expression, cis‐prediction was performed to identify lncRNA‐mRNA pairs with genome locations within 10 kb. The blat tool was used for trans‐prediction, and sequences of lncRNAs and mRNAs (3'‐UTR) were compared to screen lncRNA‐mRNA pairs with similar sequences. The results showed that the target gene of lncRNA XR_108954.2 is *E2F2* (Figure 3).

**FIGURE 4:**
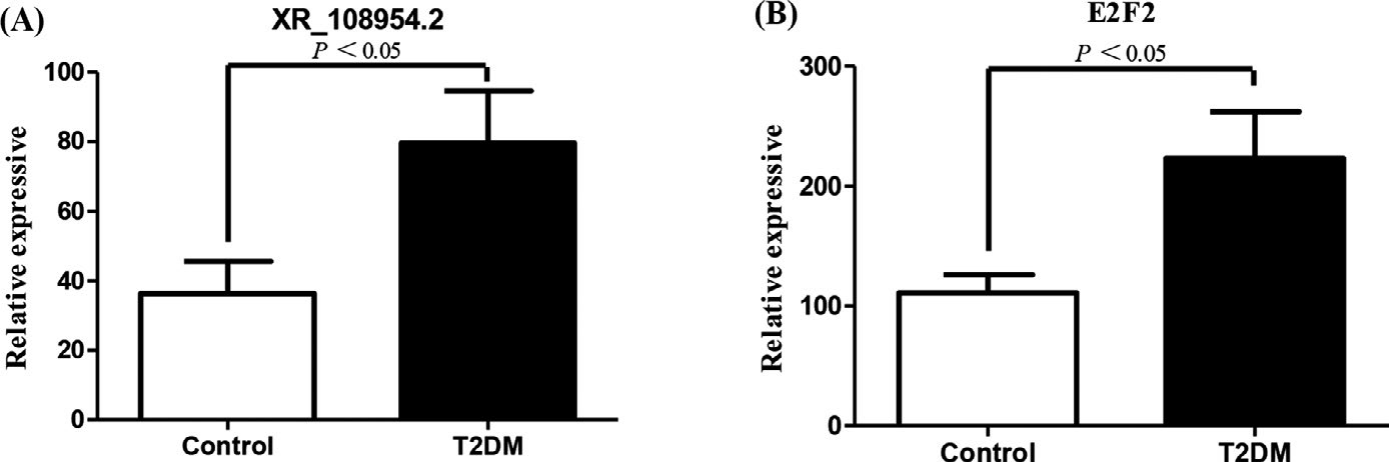
lncRNA XR_108954.2 and *E2F2* microarray expression profiling in patients with T2D and controls

The chip test results revealed that the lncRNA XR_108954.2 expression levels were higher in the T2D group as compared to the control group (79.54 ± 15.11 vs. 36.28 ± 9.24; *p* < 0.05) (Figure 4A); we also observed similar results for *E2F2* mRNA expression (223.33 ± 38.75 vs. 111.97 ± 15.10; *p* < 0.05) (Figure 4B).

### 3.5 Validation of differential expression using qRT‐PCR and ELISA assay

**FIGURE 5:**
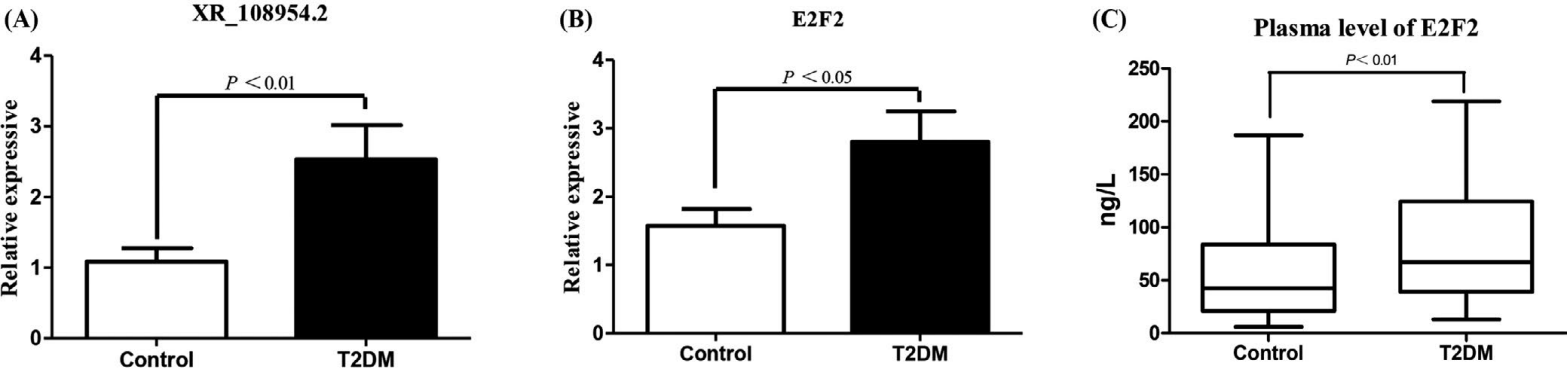
qRT‐PCR and ELISA validation of lncRNA XR_108954.2 and E2F2 expression in patients with T2D patients and controls

The expression of lncRNA XR_108954.2 and *E2F2* was verified in 100 patients with T2D and 100 healthy controls using qRT‐PCR. We found that the expression of XR_108954.2 and *E2F2* in T2D group was higher compared with that in the control group (XR_108954.2: 2.54 ± 0.48 vs. 1.09 ± 0.19, *p* < 0.01; *E2F2*: 2.80 ± 0.45 vs. 1.57 ± 0.25, *p* < 0.05) (Figure 5A, B). These data were consistent with those obtained from microarray analysis. The ELISA showed that plasma E2F2 in the T2D patients was higher than that in healthy controls (86.67 ± 5.83 vs. 57.19 ± 4.89 ng/l, *p* < 0.01) (Figure 5C).

### 3.6 Correlation between lncRNA XR_108954.2 and *E2F2* and clinical biochemical indicators

Multiple linear regression analysis was used to evaluate the correlation between lncRNA XR_108954.2 and *E2F2*. The results showed that XR_108954.2 and *E2F2* expression levels were positively correlated (*r* = 0.461, *p* < 0.01). Moreover, the correlation between biochemical indicators and lncRNA XR_108954.2 and *E2F2* was also analyzed. The multiple correlation coefficient between XR_108954.2 expression levels and glucose metabolism indicators, including FPG and GSP, was 0.387 (*p* < 0.01), and *E2F2* expression levels were positively correlated with glucose (*r* = 0.368, *p* < 0.05) and lipid metabolism indicators (*r* = 0.333, *p* < 0.05) (Table 3).

**TABLE 3:** Correlation of lncRNA XR_108954.2 and E2F2 expression with clinical characteristics

	XR_108954.2		E2F2	
	*r*	*p*	*r*	*p*
XR_108954.2	1.000	—	0.461	≤0.001
E2F2	0.461	0.000	1.000	—
Indicators of glucose metabolism				
FBG (mmol/l)	0.387	0.003	0.368	0.003
GSP (mmol/l)				
Indicators of lipid metabolism				
TG (mmol/l)	0.214	0.506	0.333	0.049
TC (mmol/l)				
HDL (mmol/l)				
LDL (mmol/l)				

### 3.7 Identification of novel T2D biomarkers

**FIGURE 6:**
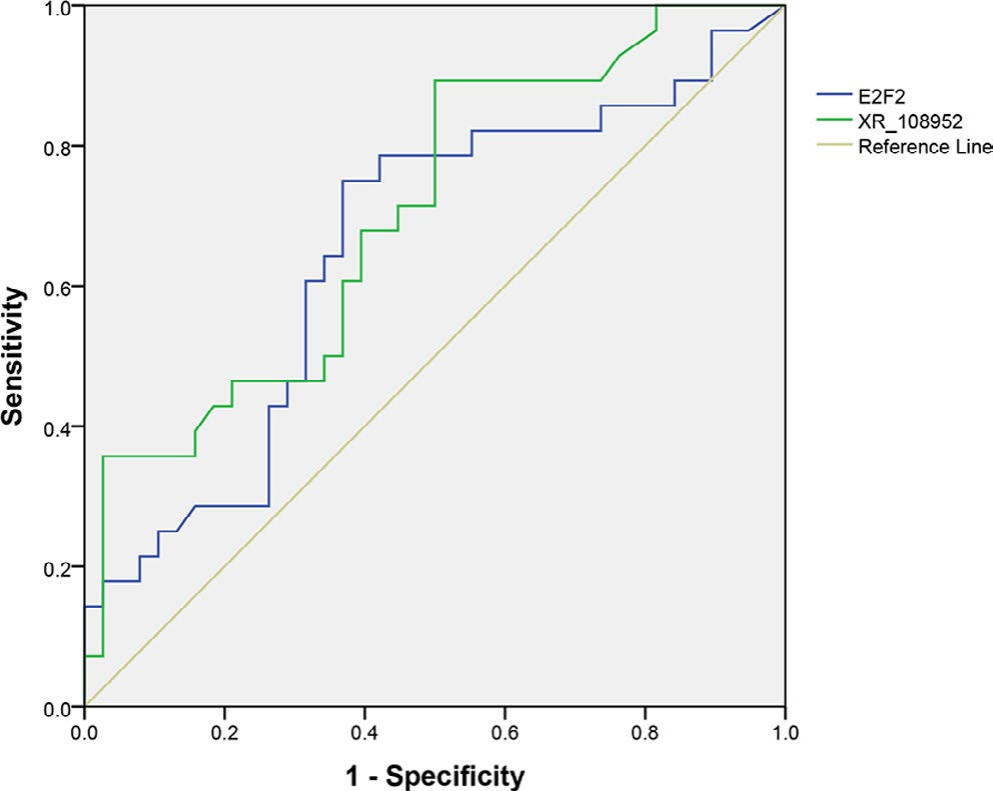
Receiver operating characteristic curve analysis for XR_108954.2 and E2F2

The diagnostic value of XR_108954.2 and *E2F2* was evaluated using ROC curve analysis. The AUC of XR_108954.2 was 0.704 (*95% CI*: 0.578–0.830, *p* = 0.05), and that of *E2F2* was 0.653 (*95% CI*: 0.516–0.790, *p* = 0.035) (Figure 6). These findings suggested that *E2F2* has better clinical significance in terms of discriminating patients with T2D from healthy controls than XR_108954.2.

## 4 DISCUSSION

lncRNAs were once considered "junk DNA" due to their non‐coding function and were thought to have accumulated during the evolution of genes.^18^ However, the rapid development of molecular biology and the application of next‐generation sequencing technologies have illustrated that lncRNAs play important roles in diverse biological functions including chromatin modification, transcriptional regulation, post‐transcriptional regulation, cellular proliferation, differentiation, and apoptosis.^19^, ^20^, ^21^, ^22^ Our study determined 68 differentially expressed lncRNAs and 74 differentially expressed mRNAs in patients with T2D.^23^ Bioinformatics analysis indicated that these lncRNAs may function in most biological processes associated with diabetes.

The role of lncRNAs in diabetes is gathering increasing amounts of attention, and numerous evidences have revealed that lncRNAs play important roles in many of the pathophysiological mechanisms.^24^, ^25^, ^26^ The lncRNAs may also serve as biomarkers in the diagnosis, prognosis, and clinical management of the disease.^27^, ^28^, ^29^ Abhishek et al. reviewed the numerous functions of lncRNA NONRATT021972 in different diabetes‐related diseases and found that NONRATT021972 is both a potential diagnostic and targeted therapy tool for diabetes‐associated diseases.^30^ Previously, lncRNA ENST00000588707.1 and TCONS_00004187 were observed to be at significantly lower levels in peripheral blood mononuclear cells of patients with T2D. The AUC values of ENST00000588707.1 and TCONS_00004187 were 0.816 (95% CI: 0.764–0.869, sensitivity 72.0%, specificity 80.3%) and 0.826 (95% CI: 0.774–0.879, sensitivity 81.6%, specificity 61.3%), respectively, which indicated that lncRNA ENST0000588707.1 and TCONS_00004187 may serve as potential biomarkers for T2D.^23^ In this study, we used bioinformatics approach to predict lncRNA‐mRNA pairs. Our results showed that *E2F2* was the target gene of lncRNA XR_108954.2 and that *E2F2* mRNA and lncRNA XR_108954.2 expression levels were higher in the T2D group. Moreover, *E2F2* and lncRNA XR_108954.2 expression correlated with glucose metabolic indicators, and *E2F2* expression correlated with glycolipid metabolic indicators. The AUC values of XR_108954.2 and *E2F2* were 0.704 (95% CI: 0.578–0.830, *p* = 0.05) and 0.653 (95% CI: 0.516–0.790, *p* = 0.035), respectively. These results indicate that XR_108954.2 and *E2F2* function in the glucose and glycolipid metabolic pathways during T2D progression and have potential diagnostic value in T2D.

E2F transcription factors are thought to play important roles in cell growth control as well as in the pathogenesis of many diseases.^31^, ^32^ Several studies have revealed that E2F proteins play important roles in the development of some mental disorders. In schizophrenia and bipolar disorder, cell cycle regulation is significantly altered and these changes include changes in the transcriptional complex controlling the expression of E2F/DP‐1 target genes critical for G2/M progression.^33^ Claire et al showed that significantly lower peripheral blood *E2F1* mRNA levels were observed in patients with depression than that in controls.^34^ Ainhoa et al showed that *E2F1*/*E2F2* compound‐mutant mice developed non‐autoimmune insulin‐deficient diabetes and exocrine pancreatic dysfunction.^35^ *E2F1* and *E2F2* transcription factors‐deficient mice developed a chronic pancreatitis‐like syndrome and became diabetic.^36^ Moreover, *E2F1* and *E2F2* transcription factors play important roles in the regulation of pancreatic exocrine cell cycle and maintenance of pancreatic beta cells.^37^ Anderson et al. pointed that about 25% of people with diabetes suffers from mental illness.^38^ China Guidelines for Type 2 Diabetes (2017) state that assessment of psychological status should be performed throughout the treatment of diabetes and that improving depression and anxiety in patients with diabetes is conducive to the control of diabetes.^39^ E2F may be a part of the underlying pathological mechanism of the comorbidity of diabetes and mental illness. In our study, we predicted that *E2F2* to be the target gene of lncRNA XR_108954.2, and a positive correlation between *E2F2* and XR_108954.2 expression levels (*r* = 0.461, *p* < 0.01) was identified. This indicates that *E2F2* expression is regulated by lncRNA XR_108954.2, which provides new significant insights for the mechanistic study of diabetes.

However, some limitations of this study need to be considered. The validity of *E2F2* and lncRNA XR_108954.2, as molecular markers of T2D, needs to be verified through additional experiments. The lack of indicators reflecting islet function means that the relationship between lncRNA XR_108954.2, *E2F2*, and islet function was not examined in this study. The correlations revealed between lncRNA XR_108954.2, *E2F2*, and lipid metabolism are preliminary and require a more comprehensive analysis for confirmation. Another limitation of this study is that target prediction was based on known chip sites, and additional mechanisms of interaction between lncRNAs and mRNAs remain to be explored.

## 5 CONCLUSIONS

In this study, 68 differentially expressed lncRNAs and 74 differentially expressed mRNAs were identified in patients with T2D. Bioinformatics analysis showed that these lncRNAs may function in the biological processes associated with diabetes. Additionally, we identified that the target gene of lncRNA XR_108954.2 is *E2F2*, which may be involved in glucose and lipid metabolism by regulating insulin secretion. Moreover, *E2F2* and lncRNA XR_108954.2 may be potential biomarkers for the diagnosis and treatment of T2D.

## CONFLICT OF INTEREST

The authors have no conflicts of interest to declare.

## INFORMED CONSENT

Signed informed consent was collected from all participants prior to the recruitment.

## Data Availability

The datasets used and/or analyzed during the current study are available from the corresponding author on reasonable request.
